# Seroprevalence and associated risk factors of contagious caprine pleuropneumonia in selected districts of South Wollo Zone Northeast Ethiopia

**DOI:** 10.1186/s12917-024-04181-x

**Published:** 2024-07-16

**Authors:** Muluwork Ashagrie, Belege Tadesse, Engidaw Abebe, Ahmed Yasine

**Affiliations:** School of Veterinary Medicine, Wollo University, Dessie, Ethiopia

**Keywords:** CCPP, cELISA, Goats, Northeastern Ethiopia, Risk factor, Seroprevalence

## Abstract

Contagious caprine pleuropneumonia (CCPP) is a severe and devastating respiratory disease of goats, which is characterized by severe serofibrinous pleuropneumonia accompanied by high morbidity and mortality. A cross-sectional study was conducted from July 2022 to January 2023 to determine the seroprevalence of CCPP and identify risk factors associated with the occurrence of CCPP in goats in five selected districts of the South Wollo Zone of the Eastern Amhara region. A total of 384 sera samples were collected from goats and examined for antibodies specific to *Mycoplasma capricolum* subspecies *capripneumoniae (Mccp)* using Competitive Enzyme-Linked ImmunoSorbent Assay (cELISA) test. Out of the total examined sera, 26 samples were positive for CCPP, giving an overall seroprevalence of 6.7% (95% CI = 6.64–9.77). A seroprevalence of 5.05%, 4.65%, 2.78%, 12.90%, and 10.77% were recorded in Ambasel, Tehuledere, Kalu, Dessie Zuria and Kutaber districts, respectively. However, there was no statistically significant difference among these five districts (*p* > 0.05). The seroprevalence of CCPP varies significantly between age groups and agroecology (*p* < 0.05). However, the seroprevalence did not vary with sex, body condition score (BCS), and flock size (*p* > 0.05). Old-aged goats (OR = 4.10) and goats found in the lowlands (OR = 5.09) were at higher risk of infection with CCPP than young-aged goats and goats found in the highlands, respectively. In conclusion, the present seroprevalence investigation indicated the occurrence of CCPP in those selected study districts of the South Wollo Zone. Therefore, appropriate control measures, including avoiding the mixing of flocks and vaccination should be designed and implemented especially in the lowland areas and older goats to reduce the further spread and magnitude of the disease.

## Introduction

Small ruminants are considered the main asset for livestock farmers in East Africa [1] and play crucial economic and cultural roles in Ethiopia [2, 3]. Goats are essential sources of cash income, meat, and milk for smallholder farmers in various agroecological zones of the country [4].

Amhara region consists of 23.7% (*n* = 7,766,661) of Ethiopia’s total goat population and has paramount economic importance [5]. Although goats represent a tremendous national resource, their productivity is below the expected due to several factors [6–8]. Diseases are the main factor that hinders the productivity of goats in Ethiopia [9]. From these diseases, contagious caprine pleuropneumonia (CCPP) causes enormous economic loss in the goat population [10].

Contagious caprine pleuropneumonia caused by *Mccp* is a severe and devastating respiratory disease that causes severe fibrinous pleuropneumonia accompanied by high morbidity and mortality in goats [11]. It is characterized by high fever, depression, weakness, loss of appetite, and associated respiratory manifestations such as cough, dyspnea, and respiratory discharges. Abortion and high mortality have also been reported in some instances [12]. The disease is a transboundary animal disease, included in the list of notifiable diseases of the World Organization for Animal Health (WOAHA) [13].

CCPP affects goats in more than 40 countries of the world, thereby posing a significant hazard to goat farming around the globe [12]. The first incidence of CCPP was reported in 1873 in Algeria, then later it was reported in South Africa, Chad, Eritrea, Ethiopia, Kenya, Mauritius, Niger, Sudan, Tanzania, Tunisia, and Uganda [14].

In Ethiopia, the presence of CCPP has been suspected since 1983 and later confirmed in 1990 by isolation and identification following an outbreak of CCPP in Ogaden, Eastern Ethiopia. The current situation of CCPP has been reported from almost all regions of Ethiopia and is a special problem for goats in lowland pastoral areas of the country [15].

The occurrence of the disease follows the introduction of an infected animal into a group of susceptible goats [10]. Mixing of different flocks and the presence of trade routes are also considered significant risk factors for exposure to CCPP [16, 17]. Various studies also showed that factors such as the inadequacy of accurate diagnostic services, shortage of vaccination against CCPP, poor management, weather conditions, and concurrent infections contribute to a wide-ranging occurrence of the disease in rural and different agroecological areas of the country [4]. The disease is transmitted by direct contact through aerosols, droplets, or nasal discharge, so close contact among goats facilitates the spread of the infection [18].

Various study reports in different areas of Ethiopia indicated that the prevalence of CCPP ranges from 5 to 78% [4, 11, 14, 15, 19–27]. Although outbreaks and high prevalence of CCPP have been reported and documented in different areas of the country, there is still no documented epidemiological information about CCPP in South Wollo. Therefore, this study was designed to estimate the seroprevalence and identify risk factors of CCPP in goats in the selected districts of the South Wollo zone.

## Materials and methods

### Study area

**Fig. 1:**
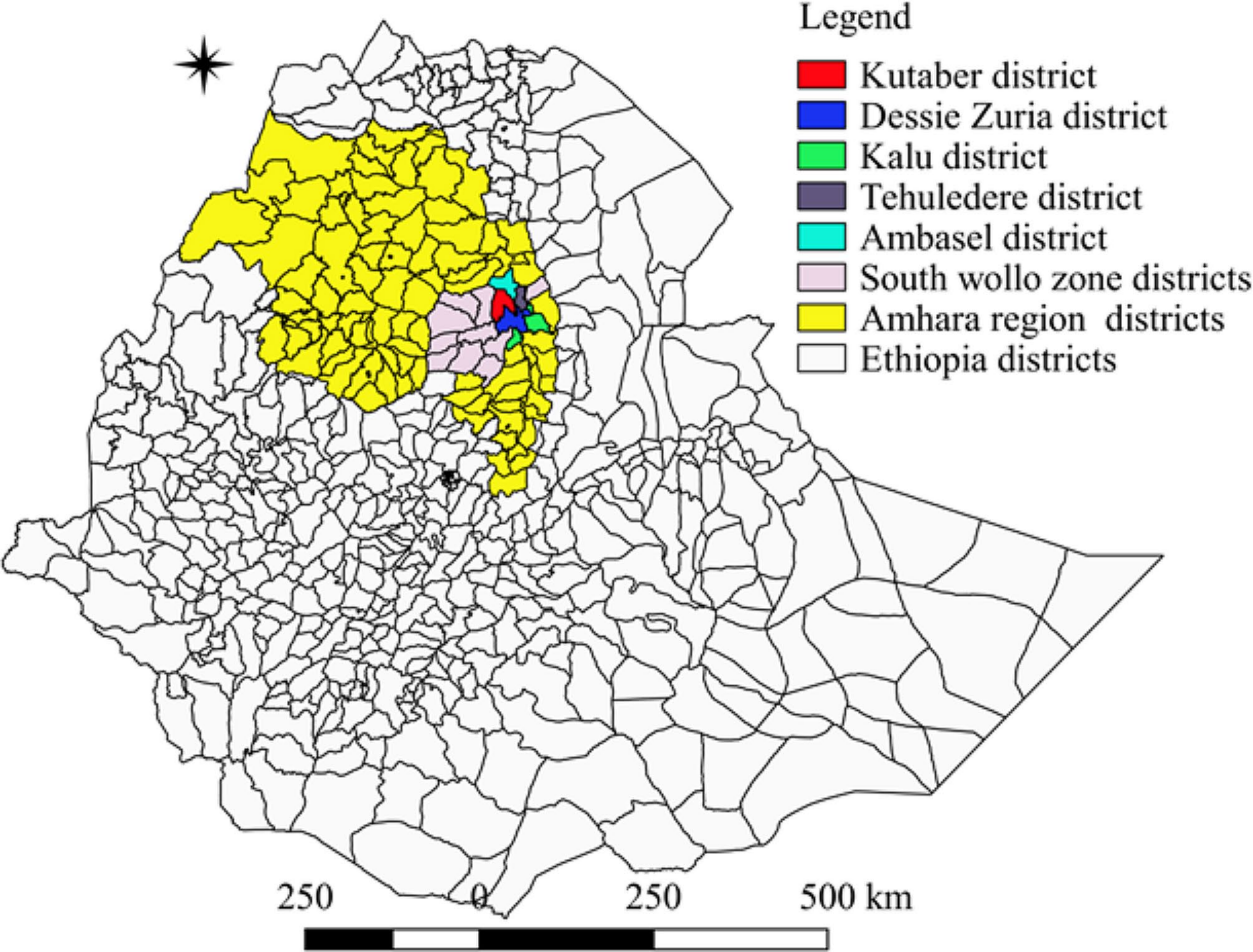
Map of the study districts done by using QGIS 2.18.28

The study was conducted in five selected districts of the South Wollo Zone, namely Ambasel, Tehuledere, Kalu, Kutaber, and Dessie Zuria (Fig. 1). A total of 1, 373,184 sheep and 826,468 goats are reared in South Wollo Zone [28]. The districts comprise lowland, midland, and highland agroecologies. Agroecology was classified into highland, midland, and lowland, according to Gari et al. [29]. The crop-livestock mixed production system is the main livelihood of the districts [30].

### Study design

A cross-sectional study was conducted from July 2022 to January 2023 to estimate the seroprevalence of CCPP and identify risk factors associated with the occurrence of CCPP in goats in the five selected districts of the South Wollo Zone of the Eastern Amhara region.

### Study population

Goats kept in the selected five districts were considered as study and/ or target population. Goats reared under a traditional extensive farming system and selected for sampling were the study animals. Goats with no history of vaccination against CCPP in the last two years, above six months old, and both sexes were selected for the study. The age of goats were determined based on dentition and considered as young (6 months to 2 years), adult (2 to 5 years), and old (> five years) [31]. The body condition was also classified as poor, medium, and sound, according to Mike, 1996 [32].

### Sample size

The sample size was estimated using the formula given by Thrusfield [33], considering a 95% confidence level, expected prevalence of 50%, and 5% desired absolute precision.

$$n = \frac{{{{1.96}^2}{P_{\exp }}(1 - {P_{\exp }})}}{{{d^2}}}$$

Where n = required sample size.

Pexp = expected prevalence.

d = desired absolute precision (5%).

Accordingly, a total of 384 serum samples were involved in the analysis. Sampling from each district was included based on the proportion of the population. Among them, 99 serum samples from Ambasel, 86 from Tehuledere, 72 from Kalu, 62 from Dessie Zuria, and 65 from Kutaber were collected. The goat population above six months age in selected PAs of each district during the study period were shown in Table 1 below.

**Table 1:** Goat population above six month age of the study districts

District	Number of Goat population above six months age
Ambasel	48,460
Tehuledere	42,250
Kalu	35,250
Dessie Zuria	30,355
Kutaber	31,810

### Sampling technique

Five districts, namely, Ambasel, Tehuledere, Kalu, Dessie zuria, and Kutaber, were selected using simple randome sampling technique (lottery method)using 23 districts in the South Wollo zone that have goat population as a sampling frame. From each selected district, two peasant associations (PAs) with no history of vaccination against CCPP in the last two years were selected using simple randome sampling via lottery method using PAs that have a goat population as a sampling frame. Within the selected PA, households/ flocks to be sampled were also selected randomly. A systematic random sampling technique was used to select goats for whole blood sample collection. The number of goats sampled from each district, PA, and household was proportional to the goat population in each district, PA, and household.

### Sample collection

Five to seven milliliters of whole blood samples were collected aseptically using sterile plain vacutainer tubes and blood-collecting needles after the site from the jugular vein of each study goat, after cleaning the site with 70% alcohol-soaked cotton. After collection, each blood sample containing vacutainer tubes was labeled with code and placed in a slant position in an ice box at room temperature for a maximum of 3–4 h until transported to the Veterinary Microbiology Laboratory of the School of Veterinary Medicine, Wollo University for serum separation.

During sampling, animal information such as breed, sex, age, body condition, signs of respiratory disease were recorded on a data collection sheet.

### Questionnaire survey

During whole blood sample collection, a data on the flock size, and other factors, including management, district, PA, production system, and agroecology were recorded by interviewing the goat owners using semi-structured questioner to identify factors that may affect the occurrence of CCPP in the districts. Agroecology was classified in to highland, midland and lowland according to Gari et al. [34]. Flock size of goats were classified as small (*< 10)*,* medium (11–20) and large (> 20) goats* [35].

### Laboratory analysis

#### Extraction of serum from the whole blood

The separation of the serum from the whole blood sample was done at the Wollo University School of Veterinary Medicine Veterinary Microbiology Laboratory. Whole blood samples were centrifuged at 1500 revolutions per minute for ten minutes, and the serum was harvested into a sterile cryogenic tube. After the serum was harvested, each cryogenic tube containing the serum was firmly closed and labeled with its respective code. Finally, each cryogenic tube was stored at − 20∘ C in the Laboratory of the School of Veterinary Medicine until further serological analysis [36].

#### Serological test

The serum samples were transported to the National Veterinary Institute (NVI), Bishoftu, Ethiopia, with a cold chain for the serological analysis. Competitive enzyme-linked immunosorbent assay (c-ELISA) was carried out for the detection of antibodies against *Mccp.* according to OIE [36] and Peyraud et al. [37]. The assay was conducted by using the C-ELISA test kit (CIRAD Montpellier, France) containing a monoclonal anti-*Mccp* antibody named MAb 4.52, precoated plates, and readymade reagents. The assay was accomplished following the manufacturer’s instructions. Briefly, 500 µls of serum samples in each cryogenic vial to be tested were diluted and mixed with 1 ml of a specific monoclonal anti-*Mccp* antibody (Mab 4.52) in a prelate (uncoated plate). Then, the homogenized contents of the prelate were transferred into the *Mccp* antigen-coated microplates and incubated for one hour at 37∘C with gentle agitation. All of the wells were washed two times with 100 ml washing solution. Anti-mouse IgG horseradish peroxidase conjugate was added to each well, and the plates were incubated for 30 min at 37∘C. Following three times washing, 100µls of substrate solution 3, 3, 5, 5-tetramethylbenzidine were added to each well and incubated for 20 min at 37∘C in a dark place. Finally, 100 µl of stop solution was added into each well with a gentle agitation, allowing a color reaction to develop. Then, the optical density (OD) of the individual reaction was measured at 450 nm with an ELISA plate reader. Results were interpreted as per the manufacturer’s instruction using the following formula: percentage of inhibition (PI) = ((OD Mab - test serum)/ (OD Mab – O conjugate)) ×100.

That is, those sera with a PI greater than or equal to 55% were considered positive for the presence of *Mccp* infection, and those sera with a PI less than 55% were considered negative [37].

The sensitivity and specificity of the used ELISA test kit were 93% and 88%, respectively. The overall true prevalence of CCPP in this study was estimated according to Thrusfield [33] based on the formula: true prevalence = (Apparent prevalence + (Specificity − 1))/(Specificity + (Sensitivity − 1)).

### Data management and analysis

The collected raw data and serology results were recorded in a Microsoft Office Excel spreadsheet. Statistical analyses were performed using STATA version 14 (Stata Corp.1985–2013) statistical software. The overall seroprevalence was calculated by dividing the number of CCPP-positive goats by the total number of goats tested and multiplied by 100. Univariable logistic regression analysis was used to estimate the odds ratios (OR) and seroprevalence. Multivariate logistic regression was employed to analyze further those risk factors having a *p*-value of ≤ 0.25 in the univariate analysis. The OR was used to measure the association and strength of association between risk factors and seroprevalence of CCPP. A *p*-value of < 0.05 was considered statistically significant.

Multicollinearity between independent variables were checked using variance inflation factor (VIF). Accordingly, the VNF between all variables was less than two, which indicates absence of multicollinearity [38].

However, since multiple logistic regression is a model that can give an odds ratio which is controlled (i.e. adjusted OR) for multiple confounders, it is used to avoid confounding of variables.

## Results

### Sero-prevalence and associated risk factors of CCPP

Out of 384 serum samples tested by cELISA, 26 (6.77% (95% CI = 6.64–9.77)) were found positive for specific antibodies against CCPP. The prevalence was highest in the Dessie Zuria district (12.9%), followed by the Kutaber district (10.77%), and the least was in the Kalu district (2.78%) (Table 2). The overall true prevalence was estimated as to be 8.21%.

The univariable logistic regression analysis showed that age, district and agroecology were statistically significant (*p* < 0.05) associated with the prevalence of CCPP. Meanwhile, sex, body condition, and flock size were not significantly (*p* > 0.05) associated with the seroprevalence of CCPP. The odds of the occurrence of CCPP in old goats was 4.10 (95% CI = 1.14–14.08; *p* value = 0.031) times higher than younger ones. The seroprevalence in the land was 5.09 (95% CI = 1.41–18.32; *p* = 0.013) times higher than that of goats in the highland (Table 2).

**Table 2:** Prevalence and univariable logistic regression analysis of host and environmental risk factors for CCPP seropositivity

Variable	Category	Number tested	No. positive (Prevalence in %)	OR (95% CI)	*P* value
Sex	M	88	6(6.82)	Ref.	
	F	296	20(6.76)	0.99(0.38–2.55)	0.984
Age	Young	123	3(2.44)	Ref.	
	Adult	143	11(7.69)	2.65(0.71–9.85)	0.147
	Old	118	12(10.17)	4.10(1.14–14.08)	0.031
BCS	Good	118	9(7.63)	Ref.	
	Medium	198	11(5.56)	0.71(0.0.29–0.1.77)	0.466
	Poor	68	6(8.82)	1.17(0.39–3.45)	0.773
District	Kalu	99	2(2.78)	Ref.	
	Tehuledere	86	4(4.65)	1.71(0.30–9.60)	0.544
	Ambasel	72	5(5.05)	1.86(0.35–9.88)	0.465
	Dessie zuria	62	8(12.90)	5.19(1.06–25.42)	0.052
	Kutaber	65	7(10.77)	4.22(0.84–21.12)	0.079
Flock size	Small	96	5(5.21)		
	Medium	205	15(7.32)	1.44(0.51–4.07)	0.496
	Large	83	6(7.23)	1.42(0.42–4.82)	0.576
Agroecology	Highland	128	3(2.34)	Ref.	
	Midland	121	10(8.26)	3.52(0.95–13.12)	0.060
	Lowland	109	13(11.93)	5.09(1.41–18.32)	0.013

The multivariable logistic regression also showed that age and agroecology were statistically significantly associated with the seroprevalence of CCPP (*p* < 0.05). The odds of occurrence of CCPP in old goats was 4.27 (95%CI = 1.17–15.64; *p*-value 0.028) times higher than in young goats. In contrast, the odds of occurrence of CCPP in low land agroecology was 6.07 (95% CI = 1.70-21.68; *p* = 0.005) times higher than that of goats in the highlands in the multivariable analysis. However, there was no statistically significant difference in the occurrence of CCPP between young and adult and also between highland and midland agroecology (*p* > 0.05) (Table 3).

**Table 3:** Multivariable logistic regression analysis of host and environmental risk factors of CCPP seropositivity

Variable	Category	OR (CI)	*P* value
Agroecology	Highland	Ref.	
	Midland	3.24(0.83–12.65)	0.091
	Lowland	6.07(1.70-21.68)	0.005
Age	Young	Ref.	
	Adult	2.56(0.68–9.60)	0.164
	Old	4.27(1.17–15.64)	0.028

## Discussion

The apparent and true overall prevalence of CCPP in the study was 6.7% (26/384) and 8.21%, respectively, with different levels of seroprevalence among the study districts. This finding was comparable with the report of Beyene [39] and Abrhaley et al. [4], who reported 6% and 8.5% prevalence of CCPP in the Dire Dawa and Western Amhara regions, respectively. In other parts of the world, a comparable prevalence of 6.5% has been reported in Tanzania [17].

The seroprevalence of CCPP in this study was lowest as compared to the prevalence reported in different parts of Ethiopia, ranging from 29 to 77% [19–21, 23, 24, 40–42]. Teshome et al. [11], Mebrahtu et al. [15]. (2017), Fasil et al. [27], and Matios et al. [26]. A higher seroprevalence of 31.5% was also reported in the Borana zone, 78.4% in the South Omo zone, Southern Ethiopia, 18.1% in Gambella, and 31.6% in Oromia, respectively. A higher seroprevalence of 44.5% and 51.8% in the Dire Dawa and Oromia regions of Ethiopia [21] and 43.9% [20] in the Tigray region of Ethiopia were also reported.

In other parts of the world, a higher prevalence than this study has been also reported in Pakistan [43], Tanzania [44], Kenya [45], and Uganda [46]. Wazir et al. [47] reported a lower seroprevalence of CCPP in Pakistan as compared to the findings of this research. The observed variation in seroprevalence of CCPP between this study and the previous studies reported from different areas might be due to differences in the husbandry practices, agroecology, vaccination history, sampling methods applied, age composition, breed, management, and sample size used. This study was conducted in all agroecology, and the prevalence is low compared to the studies mentioned above, which were mainly conducted in lowland areas. In highland areas, the prevalence becomes low as precipitating factors such as harsh environmental conditions and feed and water shortages.

On the contrary, the seroprevalence finding in this study was higher than the seroprevalence of 4.9% reported by Matios et al. [26] in Dire Dawa. The variation in the seroprevalence reports might be due to the differences in study areas’ agroecological systems, goat management and production systems, population density, and the techniques used to define the seropositivity. The traditional and extensive farming practice in this study area might spread CCPP when goats meet at communal grazing and watering areas.

The seroprevalence of CCPP in this study varies between districts. Higher prevalence was determined in the Dessie Zuria district (12.9%), and low prevalence was found in the Kalu district (2.78%). This might be a result of the presence of different animal management systems, production systems, population density, and animal movement and mixing. This was supported by the reports of Mebrahtu et al. [15], Elemo et al. [48], and Mekuria et al. [22], who reported that the seroprevalence may vary from one area to another within a country. Although there was a variation in seroprevalence between districts, there was no statistically significant difference, which agreed with the report of Teshome et al. [11] in selected districts of the Bale zone pastoral area in southeastern Ethiopia.

In this study, the seroprevalence of CCPP among age groups and agroecology revealed a statistically significant difference. Higher prevalence was found in older goats than younger ones and lowland as compared to the highland areas. This finding is in agreement with the report in Hammer and Benna Tsemay Districts of Southern Ethiopia [42], Regassa et al. [49] in selected districts of Afar Region, in selected Woredas of Afar [23], in Dire Dawa [25], in selected districts of Borana zone of Southern Oromia; in selected districts of Gambella Region [27] and in Western Amhara region [4] who reported statistically significant association among age groups and agroecology. However, Regassa et al. [49] and Eshetu et al. [39] reported the absence of difference in the prevalence of CCPP among age groups.

The high prevalence of CCPP in old goats might be due to the fact that old goats have been at risk of exposure for a more extended period than young goats to different stressor conditions like malnutrition, movement over long distances, and adverse weather conditions which can predispose the animal to the disease [46]. According to OIE [36], all age groups of goats are susceptible, and seroprevalence may be high in adult goats, but mortality is higher in young animals than in adults. This may be due to the reason that acutely infected young animals may die of CCPP before developing antibodies and are not available for testing [4]. In the lowland areas, goats may be exposed to adverse weather conditions and shortage of feed and water, movement of flocks in search of feed and water, and mixing of different flocks, which leads to the spread of CCPP, and the prevalence of CCPP will become high.

Host-related variables such as sex and body condition score of goats were not significantly associated with CCPP seroprevalence. But there was slightly higher seroprevalence in males and poor-conditioned goats as compared to females and good-conditioned goats, respectively. This is in agreement with the findings reported by Mekuria et al. [22], Bekele et al. [23], and Atim [46] but disagrees with the findings of Hadush et al. [20] and Mbyuzi [44], who reported higher seroprevalence of CCPP in female goats than goats.

Slightly higher seroprevalence was detected in medium (7.32%) and large size (7.23%) flocks as compared to small (5.21%) flocks. This agrees with the report of Parray et al. [50], who reported that large flocks were more affected by CCPP than smaller flocks. This might be attributed to the fact that large flock-size populations lead to overcrowding, which favors the spread of the disease within the flock by increasing the contact rates between infected and susceptible goats. On the contrary, various studies reported an insignificant impact of flock size on the CCPP prevalence [11, 45].

This study is very important to know the status of CCPP and understand the factors that may precipitate the occurrence of CCPP in the study districts. The produced information could be used as a baseline information for the implementation of feasible control methods.

In this study, the knowledge, attitude and practice of the goat owners towards CCPP was not assessed and this was the limitation of the study.

## Conclusion

The overall seroprevalence in this study was slightly low. The seroprevalence was higher in the Dessie Zuria district and lowest in the Kalu district. The seroprevalence of CCPP significantly varies between age and agroecology, and higher seroprevalence was found in old goats and goats in the lowland areas. However, sex, flock size, BCS, and districts did not show a statistically significant difference in the seroprevalence of CCPP. Therefore, this study indicated the occurrence of CCPP in the selected study districts of South Wollo Zone, which seeks the implementation of appropriate and integrated control measures. Appropriate control measures, including vaccination, avoiding the mixing of flocks, and movement control, should be implemented by CCPP to reduce the further spread of the disease and to prevent the infection of new herds. The responsible veterinary authority should apply regular monitoring and surveillance of the status of the disease.

## Data Availability

The datasets generated and/or analyzed during this study can be available from the corresponding author upon reasonable request.
